# Supplementary material for: Developing a Compost Quality Index (CQI) Based on the Electrochemical Quantification of Cd (HA) Reactivity
Source: Molecules. 2023 Feb 3;28(3):1503. doi: 10.3390/molecules28031503 (PMC9919838; doi:10.3390/molecules28031503)
Supplement: Supplementary file 1 [file molecules-28-01503-s001.zip › molecules-2163623-supplementary.pdf]

Supplementary Material

Data obtained through acid-base titrations following the methodology proposed by López et al. (2021) [16].

Table S1: Abundance of deprotonated groups at pH 7.0 ($Q_{pH7.0}$) and the abundance of acid sites of the extracts HA, FA and DOM (M_T, $M_{T,HS}$ and $M_{T,DOM}$). The abundance of acid sites of the extracts was calculated using Equations S3 and S4.

	Parameters		CVA*	CVDW*	CA	CLW*	CUW*	CDDW*	CSS*	FLW
	$Q_{pH\,7.0}$	(mmol g_C^{-1})	4.18	4.53	4.92	4.23	5.39	5.43	3.57	3.74
HA	M_T	(mmol g_C^{-1})	6.65	7.13	8.28	7.14	8.95	8.73	6.32	5.68
	$M_{T,HS}$	(mol $kg_{compost}^{-1}$)	0.12	0.1	0.09	0.14	0.1	0.08	0.09	0.18
	$Q_{pH\,7.0}$	(mmol g_C^{-1})	8.10	10.2	10.9	8.46	9.12	9.41	11.0	7.09
FA	M_T	(mmol g_C^{-1})	11.3	14.1	16.6	12.9	12.5	13.7	18.5	10.6
	$M_{T,HS}$	(mol $kg_{compost}^{-1}$)	0.01	0.03	0.02	0.03	0.02	0.02	0.04	0.04
	$Q_{pH\,7.0}$	(mmol g_C^{-1})	4.59	2.18	3.31	2.36	1.85	5.65	2.84	2.55
DOM	M_T	(mmol g_C^{-1})	9.89	4.32	6.7	5.63	3.92	11.3	8.38	5.92
	$M_{T,DOM}$	(mol $kg_{compost}^{-1}$)	0.22	0.62	0.10	0.97	0.43	0.26	1.17	1.09

* Data from López et al. (2021) [16]

The carbon content of each extract C_{HS} and C_{DOM} were calculated using equations (S1) and (S2), for the HS and DOM, respectively:

$$C_{HS} = \frac{C}{100} \times Y \qquad \text{(S1)}$$

$$C_{DOM} = \frac{DOC}{\frac{m_{compost}}{V}} \qquad \text{(S2)}$$

where, *C* is the carbon content of the HS (% w/w), *Y* is the yield of the extractions (w/w, g kg^{-1}), *DOC* is the concentration of dissolved organic carbon (mg L^{-1}) and $m_{compost}/V$ is the mass of compost *per* volume of solution used to prepared the equilibrium solution (50 $g_{compost}$ L^{-1}).

Table S2: Carbon content of the extracts HA, FA and DOM and yield (Y) of the extractions of HA and FA. The carbon content of each extract was calculated using Equations S1 and S2.

Sample	HA			FA			DOM	
	C (%)	Y (g kg^{-1})	C_{HA} (g_C $kg_{compost}^{-1}$)	C (%)	Y (g kg^{-1})	C_{FA} (g_C $kg_{compost}^{-1}$)	DOC (mg L^{-1})	C_{DOM} (g_C $kg_{compost}^{-1}$)
**CVA**	53.5*	33.4*	17.9	49.7*	2.27*	1.14	111*	2.22
**CVDW**	52.7*	19.0*	13.9	39.8*	4.73*	1.87	717*	14.3
**CA**	53.3	19.9*	10.6	45.8*	2.67*	1.22	75.2*	1.5

**CLW**	55.2*	34.9*	19.3	35.4*	7.00*	2.48	861*	17.2
**CUW**	54.5*	21.1*	11.5	40.8*	3.33*	1.35	548*	11
**CDDW**	50.6*	26.4*	9.62	38.4*	4.20*	1.61	115*	2.31
**CSS**	58.5*	23.8*	13.9	40.9*	4.84*	1.97	698*	14
**FLW**	57.5	53.6	30.9	36.9*	10.9*	4.02	921*	18.4

* Data from López et al. (2021) [16]

The abundance of the acid sites, $M_{T,HS}$ and $M_{T,DOM}$ (expressed in mol $kg_{compost}^{-1}$) were calculated attending to the values of M_T from each extract (Table S2, expressed in mmol g_C^{-1}) and to C_{HS} *or* C_{DOM} (Table S3, expressed in g_C $kg_{compost}^{-1}$), using equations (S3) and (S4).

$$M_{T,HS} = M_T \times C_{\mathrm{HS}} \quad \text{(S3)}$$

$$M_{T,DOM} = M_T \times C_{DOM} \quad \text{(S4)}$$

Table S3: Identification of the compost and fertiliser samples characterized in the present work.

SAMPLE	COMPOSTING METHODOLOGY	RAW MATERIAL
Compost of urban waste **(CUW)**	Tunnel composting	Selective collection of household food waste, food waste from restaurants, canteen, markets, fairs, festivities, pilgrimages and events, green waste from cemetery and household
Vermicompost of algae **(CVA)**	Vermicomposting	60% animal waste and 40% vegetable remains (fruits and algae), digested by *Eisenia foetida* earthworms.
Vermicompost of domestic waste **(CVDW)**		Green waste (flowers, leaves, grass, fruit peels) and brown waste (straw, dry leaves, dry grass), digested by *Eisenia foetida* or *Lumbricus rubellus* earthworms.
Compost of livestock waste **(CLW)**	Pile composting	100% animal waste (a mixture of 5% sheep manure without straw, 25% chicken manure and 70% pig manure)
Compost of algae **(CA)**		60% animal waste and 40% vegetable remains (fruits and algae).
Compost of sewage sludge **(CSS)**		Forestry waste, sludge from urban wastewater treatment and sludge from local effluent treatment.
Domestic compost of domestic waste **(CDDW)**	Domestic Composting	Green waste (flowers, leaves, grass, fruit peels) and brown waste (straw, dried leaves, dried grass).
**ORGANIC FERTILISER**		
Fertiliser of livestock waste **(FLW)**	-	100% animal waste (chicken manure). Mixture subjected to a high temperature to eliminate pathogens.

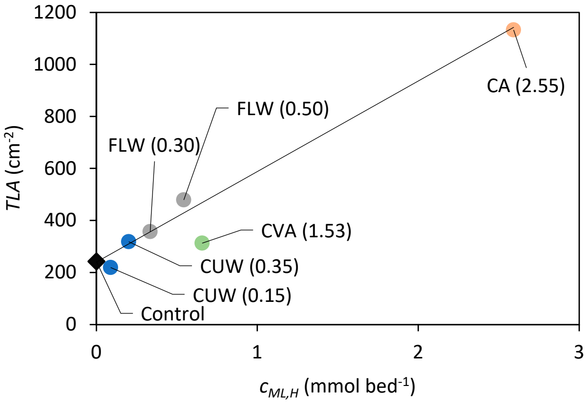

Figure S1: Correlation between the total leaf area (*TLA*) of lettuce from a crop field assay and the parameters $c_{ML,H}$ (0.30 μmol L^{-1}) obtained from metal titrations of the HA extracts.

Table S4: Data from C/N molecular ratio of the compost and fertiliser samples. Adapted from [3]

CVA	CVDW	CA	CLW	CUW	CDDW	CSS	FLW
12.5	9.2	9.97	12.8	12.6	10.3	8.6	13.2
